# Highly luminescent near-infrared-emitting gold nanoclusters with further natural etching: photoluminescence and Hg^2+^ detection

**DOI:** 10.1186/1556-276X-7-348

**Published:** 2012-06-27

**Authors:** Shuhong Lian, Dehong Hu, Changchun Zeng, Pengfei Zhang, Songhao Liu, Lintao Cai

**Affiliations:** 1 Lab of Photonic Chinese Medicine, College of Biophotonics, South China Normal University, Guangzhou, Guangdong, 510631, China; 2 Shenzhen Key Laboratory of Cancer Nanotechnology, CAS Key Lab of Health Informatics, Institute of Biomedicine and Biotechnology, Shenzhen Institutes of Advanced Technology, Chinese Academy of Sciences, Shenzhen, 518055, China

**Keywords:** Near-infrared-emitting gold nanoclusters, Natural etching, Highly luminescent, Hg^2+^, detection

## Abstract

Highly luminescent near-infrared (NIR)-emitting gold nanoclusters (Au NCs) protected by glutathione with ultra-small size were prepared at high temperature following with a further natural etching at room temperature. The optical and surface properties of Au NCs were monitored by ultraviolet–visible and photoluminescence (PL) spectra, high-resolution transmission electron microscopy, and electrospray ionization mass spectrometry. The diameter of the etched Au NCs was reduced to approximately 1.35 nm with 30 % PL quantum yield. Interestingly, the PL of Au NCs was decreased obviously by the addition of Hg^2+^ and increased by the addition of Pb^2+^ at certain concentration. Our preliminary results illustrated that the highly luminescent NIR-emitting Au NCs would be an alternative probe for the detection of heavy metal ions in water and environmental monitoring.

## Background

Gold nanoclusters (Au NCs) have received extensive attention because of their interesting optical properties [1-3], fluorescence [4,5], magnetism [6], and redox properties [7], as well as their potential applications in many fields such as optics [8-10] and catalysis [11,12]. To synthesize these highly fluorescent Au NCs, several methods have been developed. A method of synthesizing Au NCs is based on the reducing Au^3+^ ions in the presence of thiol ligands. For example, Dyer et al. exemplified the application of this approach by synthesizing Au NCs encapsulated by polyamidoamine dendrimers [13]. Ying and coworkers developed a new method of Au NC synthesis using protein bovine serum albumin (BSA) as sole reduction agent at high pH [14]. The other method of synthesizing Au NCs is based on the etching process using polymers. For example, Duan and Nie report a ligand-induced etching process for preparing highly fluorescent and water-soluble Au NCs [15]. Jin et al. found that gold nanoparticles could be etched to produce ultrasmall clusters under reflux at high temperature [16]. Inspired by this discovery, we wonder whether the etching method can be applied to using thiol ligands other than polymers.

Glutathione (GSH), the most abundant low-molecular weight thiol with naturally defined chemical components and structures, has been extensively used as ligands to induce the nucleation and growth of nanocrystals based on biomineralization or as templates to synthesize nanocrystals with well-defined morphologies [17]. Au NCs, protected with GSH, have been well studied [17,18]. Recently, Shichibu et al. discovered that some GSH-protected clusters could be etched by GSH, which generated GSH-protected Au_25_ as the main product [3,19]. However, the quantum yield of these reported gold nanoclusters is relatively low. Hence, a method of creating Au NCs with monodispersed, ultra-small size, high quantum yield and near-infrared (NIR) emission would be extremely valuable for extending their fundamental properties and investigation of a wider range of applications.

In this work, we reported a novel synthetic method, based on the capability of a common commercially available thiol ligands, GSH, for the preparation of ultra-small size highly luminescent Au NCs at high temperature following with a further natural etching at RT with NIR emission (*λ*_em max_ = 650 nm, quantum yield (QY) ≈ 30 %). Our process is similar to the biomineralization behavior of organisms in nature: sequestering and interacting with organisms, followed by providing scaffolds for the minerals formed. The optical and structural properties of the etched Au NCs were performed by ultraviolet–visible (UV–vis) and photoluminescence (PL) spectroscopies, high-resolution transmission electron microscopy (HRTEM), and electrospray ionization mass spectrometry. The PL of the etched Au NCs was decreased obviously by the addition of Hg^2+^ and increased by the addition Pb^2+^ at certain concentration. Such etched Au NCs have great potential application in heavy metal ion detection in water and environmental monitoring.

## Methods

### Materials

Chloroauric acid trihydrate (HAuCl_4·_3H_2_O, 99.99 %) was purchased from Alfa Aesar (Ward Hill, MA, USA). Sodium tetrahydroborate (NaBH_4_, 99 %) and GSH in the reduced (GSH, 98 %) form were obtained from ACROS ORGANICS (NJ, USA). All other chemicals used in this study were of analytical reagent grade and used without further purification. A JL RO100 Millipore-Q Plus water purifier (Millipore Co., Billerica, MA, USA) supplied deionized water with a resisitivity of 18.25 MΩ·cm.

### Synthesis of Au NCs

Au NCs were synthesized using the reported protocol, with a few modifications [17]. To a 50 mL methanol solution (0.5 mM) of HAuCl_4·_3H_2_O, 1.0 mM GSH (1:2 molar ratio, the total volume of methanol was 50 mL) was added. The mixture was cooled to 0 °C in an ice bath for 30 min. An aqueous solution of NaBH_4_ (0.2 M, 12.5 mL), cooled to 0 °C, was injected rapidly into the above mixture under vigorous stirring. The mixture was allowed to react for another hour. The resulting precipitate was collected and washed repeatedly with methanol through centrifugal precipitation. Finally, the Au NCs precipitate was dried and collected as a dark brown powder.

### Etching of Au NCs with GSH

The above clusters (1 mg) were dissolved in 1 mL of deionized water that contained 0.0125 mol of GSH. The mixture reacted at 65 °C ± 5 °C for 24 h at 500 rpm in the reaction container then the supernatant was put in the dark at room temperature for 5 to 8 days. The products were precipitated by added methanol (volume ratio 1:1), then the precipitate was collected and washed repeatedly with methanol through centrifugation. Finally, the products were dried by a vacuum drier and can be dissolved in water easily.

### High-resolution transmission electron microscope

Au NCs dissolved in deionized water were used directly. HRTEM specimens were prepared by drop costing one or two drops of the solutions onto an ultra-thin carbon-coated film supported on a copper grid. Bright field HRTEM images were acquired with an electron microscope operated at 200 kV (JEM-2100HR, JEOL, Japan). Typical magnifications of the images were × 200,000 and × 400,000. Moreover, energy-dispersive X-ray spectroscopy (EDS) was taken three points a sample at the same time using the affix of JEM-2100HR IET250.

### Optical absorption and photoluminescence spectroscopy

UV–vis absorption of the Au NCs was recorded in aqueous solution at ambient temperature using a PerkinElmer Lambda 25 spectrophotometer (PerkinElmer, Waltham, MA, USA). The PL spectra were obtained by a spectrofluorometer (F900, Edinburgh Instruments Ltd., West Livingston, UK). The sample solutions were not deaerated prior to the measurement. The cluster concentrations for the PL measurement were typically <10 μM, where the intensities increase linearly with the cluster concentrations. The PL quantum yield was determined according to [15].

### Electrospray ionization mass spectrometry

Mass spectrometric studies were conducted using an electro spray (electrospray ionization mass spectrometry (ESI-MS)) system (LC-6A, Japan). Samples with a concentration lower than 10 ppm, taken in deionized water, were electrosprayed at a low rate of 10 μL/min and an ion spray voltage of 5 kV.

### Assay of metal ions

A series concentration of aqueous Hg^2+^ was added to cluster solutions one by one and equilibrated at room temperature for 10 min prior to measurement of the PL with an excitation wavelength at 470 nm. All other ions (Pb^2+^, Cu2+, Ca^2+^, Mg^2+^, K^+^, and Na^+^) tested in interference studies using the same method.

### Safety considerations

As Hg^2+^ and Pb^2+^ are highly toxic and have adverse effects on human health, all experiments involving heavy metal ions should be performed with protective eyeglasses, chemical safety gloves, and protective clothing to prevent skin exposure. The waste solutions containing heavy metal ions should be collectively reclaimed to avoid polluting the environment.

## Results and discussion

### The optical property of Au NCs and its etching effect

**Figure 1:**
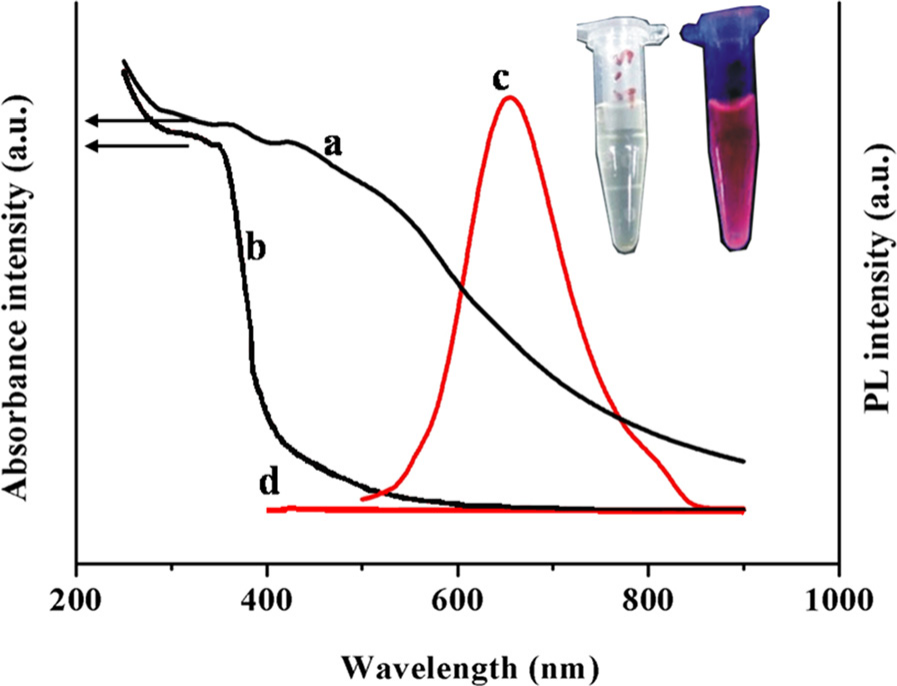
**UV–vis and PL spectra.** UV–vis (a and b) and PL spectra (c and d) of Au NCs before etching (a and d) and after etching (b and c). The photographs of Au NCs after etching are shown as an inset. The clear solution is under visible light, and the red emission is under a UV lamp. The fluorescence emission spectra were measured at the excitation wavelength of 470 nm.

The well-defined GSH-protected Au NCs were further etched by GSH in different mass ratio of Au NCs and GSH in aqueous solution at various temperatures. The as-prepared and etched Au NCs were detected by UV–vis absorbance and PL spectroscopies. As shown in Figure 1a, the absorption spectrum of Au NCs exhibited a strong and broad absorption band at approximately 500 nm, which indicated that the particle size of Au NCs was smaller than 2.5 nm [20]. As shown in Figure 1b, the absorption spectrum of the etched Au NCs has no absorption band after natural etching at RT. The spectral behavior of the Au NCs is in sharp contrast to that of the etched Au NCs. The reaction of etching leads to a remarkable depression of the UV–vis absorption, which suggests that a transformation of the original clusters into extremely small clusters has occurred [21]. Au NCs show no emission (Figure 1d), and the strong emission peak of the etched Au NCs was observed at approximately 650 nm after natural etching at RT (Figure 1c). The etched Au NC solution was clear under visible light and an intense red PL under UV light (365 nm). The maximum PLQY of the as-prepared etched Au NCs was 30 %, which was higher than PL BSA-Au NCs reported by Ying's group [14]. The PL intensity can be stable for more than half a year at room temperature when the clusters were freeze dried.

### Time-dependent-characterized optical spectra

**Figure 2:**
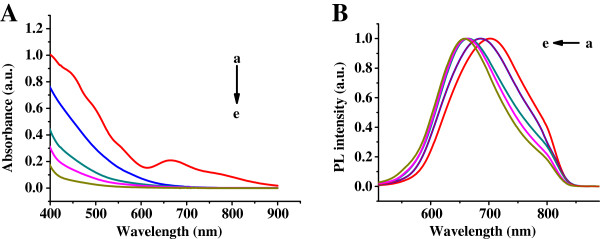
**Time-dependent UV–vis (A) and FL (B, normalized) spectra.** Taken during the core etching reaction of Au NCs with GSH. (a) 1, (b) 2, (c) 3, (d) 5, and (e) 10 days.

The optical properties of the etched Au NCs were monitored by UV–vis and PL spectra at different time scales at room temperature. As shown in Figure 2A, the absorption spectrum of the etched Au NCs exhibited a strong and broad absorption band at approximately 670 nm after natural etching at RT for 1 day, and the absorption spectrum of the etched Au NCs has no absorption band after natural etching at RT for 2 to 10 days. There is no feature at the spectrum of the etched Au NCs with relatively lower absorbance compared to the un-eched Au NCs. The result suggests that the Au NC has transformed into smaller etched Au NCs [17,22]. As shown in Figure 2B, the maximum emission peak of the etched Au NCs was observed at approximately 720 nm after natural etching at RT for 1 day, and the maximum emission peak of the etched Au NCs was blueshifted to approximately 650 nm with an obvious change of PL spectrum profiles after natural etching at RT for 2 to 10 days.

### The structural properties of Au NCs characterized by HRTEM

**Figure 3:**
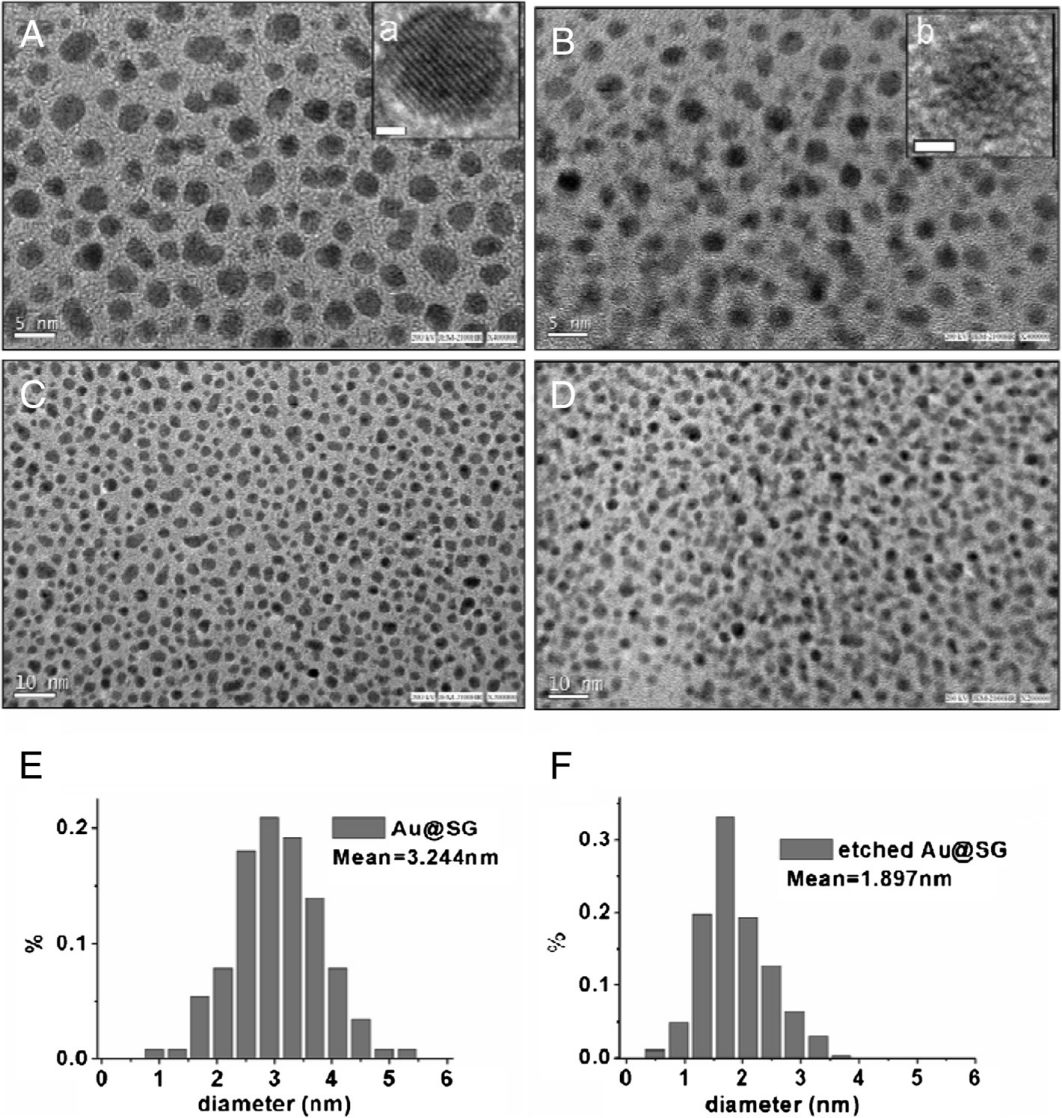
**HRTEM images of directly synthesized (A and C) and etched (B and D) Au NCs.** The small bars are 1 nm in inset a and b, 5 nm in A and B, and 10 nm in C and D. The diameter dispersion in **E** and **F** represents the distributions calculated from the corelDRAW date.

HRTEM images of the as-prepared and etched Au NCs for 5 days, as shown in Figure 3, indicate that the particles are well-dispersed. The diameter of the dispersed as-prepared Au NCs is approximately 2 to 4 nm, with a mean diameter of 3.24 nm, and the diameter of the etched Au NCs is approximately 1.0 to 2.5 nm, with a mean diameter of 1.89 nm.

EDS analysis was employed to determine the chemical identities of the etched Au NCs (Table 1). EDS measurements were conducted to confirm the presence of the GSH at the surface of the etched Au NCs. The element analysis of the etched Au NCs with EDS indicates the presence of Au from the etched Au NCs and also C, N, O, and S from the GSH molecules. The high sulfur percentage confirms an existence of a significant amount of GSH molecule at the surface of the etched Au NCs; the ratio of S/Au was determined to be approximately 1.4.

**Table 1:** Element analysis of the etched Au NCs with EDS

**Element**	**Weight percentage (wt.%)**	**Atomic percentage (at.%)**
C	13.48	49.45
N	3.80	11.95
O	1.00	2.76
S	15.26	*20.97*
Au	66.47	*14.87*
Total	100.00	100.00

### ESI-MS analysis of the clusters

**Figure 4:**
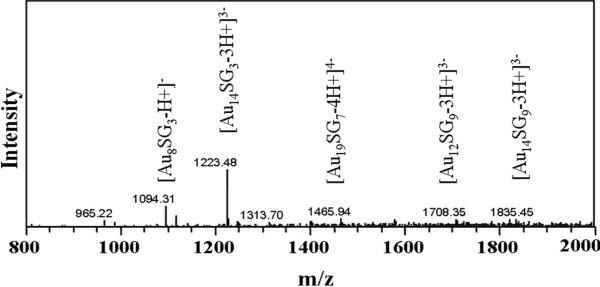
ESI-MS spectra of the etched Au NCs.

Figure 4 shows the negative-ion ESI-MS spectra of the etched Au NCs in detail. The mass range available is not enough to see the singly charged cluster. Besides, orthogonal ESI-MS does not give intact cluster species. Hence, we could not see the intact Au core. However, we see the presence of -SG, and small fragments of the Au NCs. The peaks at m/z 1094.31, 1223.48, 1465.94, 1708.35, and 1835.45 are assigned to [Au_8_SG_3_-H+]^−^, [Au_14_SG_3_-3H+]^3−^, [Au_19_SG_7_-4H+]^4−^, [Au_12_SG_9_-3H+]^3−^, and [Au_14_SG_9_-3H+]^3−^, respectively. The result shows that Au_14_ is the major core. Although the data shows a few impurities, it did not influence the application.

### Effect of Hg^2+^ on the etched Au NCs

**Figure 5:**
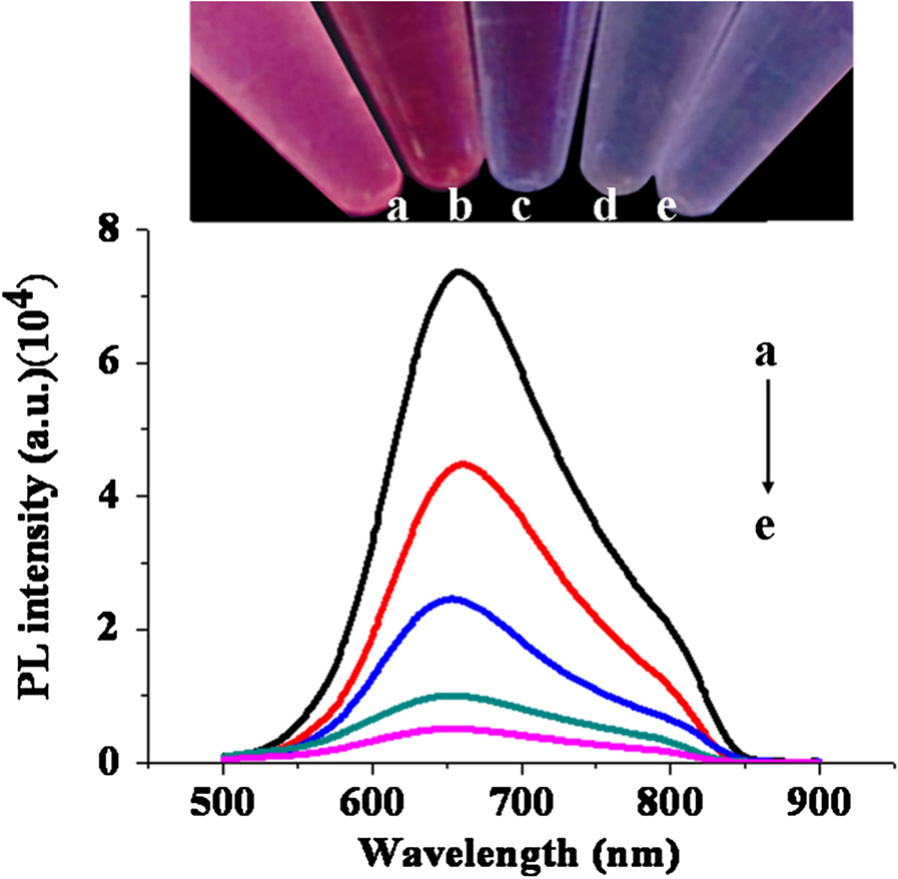
**FL response.** Of the etched Au NCs (etching for 2 days) upon addition of Hg^2+^ ions ((a) 0 nM, (b) 1 nM, (c) 100 nM, (d) 10 μM, and (e) 1 mM); FL spectra (down) and photo of the samples taken under UV lamp (up).

The effect of Hg^2+^ on the PL spectra was investigated. As shown in Figure 5, with the increasing concentration of Hg^2+^, the PL intensity of the etched Au NCs (etching for 2 days) was decreased obviously. Meanwhile, the spectral wavelength maximum was blueshifted 5 to 10 nm, which indicates the change of surface states of the clusters since Hg^2+^ ions bind onto the particle surface [23]. These values implied that the etched Au NCs have high sensitivity and reproducibility for Hg^2+^ assay. It may be used to detect trace toxicity metal ions in water due to the high PLQY.

### Interference studies

**Figure 6:**
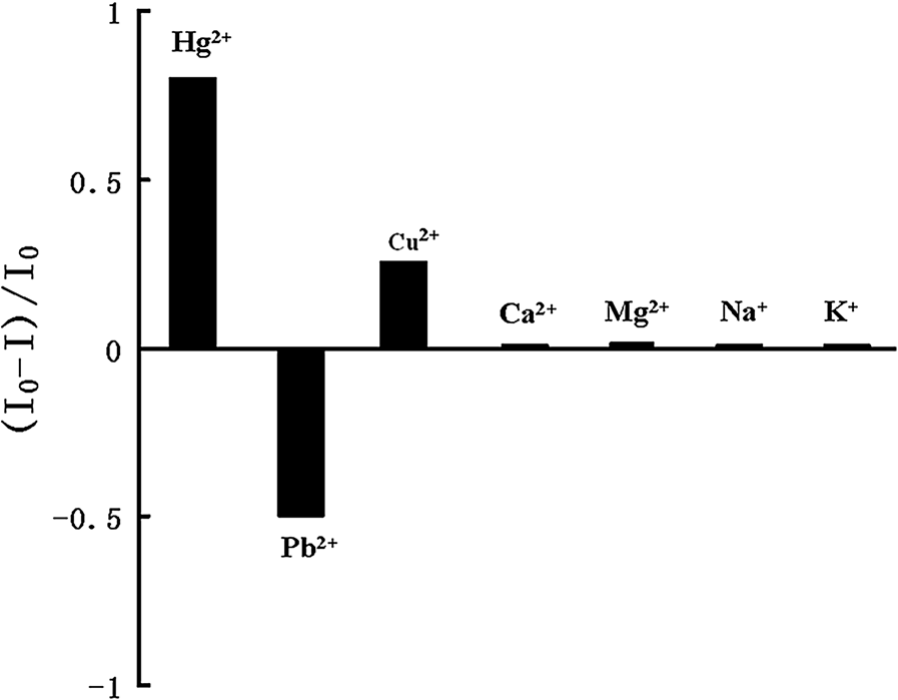
**Effect of different solutions on the**$\frac{I_{0}-I}{I_{0}}$**.**

PL quenching of the etched Au NCs was studied with the addition of the same concentration of different ion solvents (Pb^2+^, Ca^2+^, Cu^2+^, Mg^2+^, K^+^, and Na^+^) (0.2 mM). As shown in Figure 6, Pb^2+^ led to decreases in the $\frac{I_{0}-I}{I_{0}}$ value, whereas the other ions exhibited no significant effects under identical conditions. These features contributed to the excellent selectivity toward Hg^2+^ and Pb^2+^, rendering the etched Au NCs suitable for the heavy metal ions detection in water and environmental monitoring.

### Possible reaction mechanisms

A PL quenching was observed in the presence of Hg^2+^, which could be explained in terms of electron transfer between the excited etched Au NCs to the Hg^2+^[24]. The mechanism can be explained using a photoinduced electron transfer process. First, the etched Au NCs and Hg^2+^ ion form a complex. Second, the electrons within the etched Au NCs are firstly excited to the excited state under photo-irradiation. Third, Hg^2+^ can directly intercept one of the charge carriers and is reduced to Hg^+^, which can disrupt the radiative recombination of the holes and the excited electrons and quench the PL of the etched Au NCs. This assumption is supported by the following facts: In this test, GSH was used in competition with Au NCs for Hg^2+^. The PL of Au NCs was not quenched after the addition of the intermixture (1 mM Hg^2+^, 0.1 mM GSH). In a control experiment, GSH showed no influence on the PL of Au NCs in the absence of Hg^2+^, which confirmed that the complex formation occurred between GSH and Hg^2+^. Based on the finding, we concluded that the complex of Au NCs and Hg^2+^ and a photoinduced electron transfer process were the primary mechanism for PL quenching.

The reason of enhanced fluorescence in the presence of Pb^2+^ was very complicated. The spatial confinement of free electrons in the etched Au NCs results in discrete and size-tunable electronic transitions, leading to molecular-like properties. Therefore, the luminescent mechanism for the etched Au NCs would involve relaxed luminescence across the HOMO-LUMO gap [25]. When the Pb^2+^ was added into the etched Au NCs, HOMO-LUMO gap would be decreased [26]. Therefore, the enhanced luminescence of the etched Au NCs was observed. Due to the complicated reaction process, the precise luminescence enhancement mechanism remains unclear, and the detail reaction mechanism was under the way.

## Conclusions

We report herein a further etching and characterization of well-defined Au NCs protected by GSH ligands. The spectroscopic measurement of these clusters revealed that a new method of etching Au NCs was developed, and high PLQY clusters were obtained. After etching, the diameter of the Au NCs decreased to approximately 1.35 nm with 30 % PLQY. These features contributed to the excellent selectivity toward Hg^2+^ and Pb^2+^, rendering the GSH-Au NCs highly suitable for the analysis of environmental samples containing other metal ions.

## Competing interests

The authors declare that they have no competing interests.

## Authors' contributions

ShL and PZ carried out the preparation of Au NCs and drafted the manuscript. DH carried out the assay of ion experiments and modified the manuscript. CZ carried out the HRTEM experiments. SoL took part in the ESI-MS and HRTEM experiments. LC conceived of all the study and participated in its design and coordination. All authors read and approved the final manuscript.

## Authors' information

The study was conducted by the research team of the Shenzhen Institutes of Advanced Technology, Chinese Academy of Sciences. Prof. LC is the head of the team, and the team carries out the world's leading research on a range of problems of biomedical nanotechnology (nanotechnology, functional materials, surface analytical chemistry, optoelectronics, polymer science, and electrochemistry). Our aim is dedicated to studying multifunctional and nanostructured composite materials, providing highly sensitive and selective detection methods through molecular probes for medical imaging and molecular diagnosis in nanoscale and single molecular level, and exploring new device concepts and self-assembly techniques for the development of biomedical nanodevices and sensors for biosensing, environmental monitoring, information processing, energy utility, and other applications.
